# Identification of Patient Perceptions That Can Affect the Uptake of Interventions Using Biometric Monitoring Devices: Systematic Review of Randomized Controlled Trials

**DOI:** 10.2196/18986

**Published:** 2020-09-11

**Authors:** Alexander Perlmutter, Mehdi Benchoufi, Philippe Ravaud, Viet-Thi Tran

**Affiliations:** 1 Department of Epidemiology Mailman School of Public Health Columbia University New York, NY United States; 2 School of Global Public Health New York University New York, NY United States; 3 UMR1153 (METHODS team) Centre de Recherche en Epidemiologie et StatistiqueS Institut national de la santé et de la recherche médicale Paris France

**Keywords:** systematic review, patient perceptions, biometric monitoring device, randomized controlled trials, accelerometer, pedometer, ecological momentary assessment, electrochemical biosensor, adoption, uptake, real-world

## Abstract

**Background:**

Biometric monitoring devices (BMDs) are wearable or environmental trackers and devices with embedded sensors that can remotely collect high-frequency objective data on patients’ physiological, biological, behavioral, and environmental contexts (for example, fitness trackers with accelerometer). The real-world effectiveness of interventions using biometric monitoring devices depends on patients’ perceptions of these interventions.

**Objective:**

We aimed to systematically review whether and how recent randomized controlled trials (RCTs) evaluating interventions using BMDs assessed patients’ perceptions toward the intervention.

**Methods:**

We systematically searched PubMed (MEDLINE) from January 1, 2017, to December 31, 2018, for RCTs evaluating interventions using BMDs. Two independent investigators extracted the following information: (1) whether the RCT collected information on patient perceptions toward the intervention using BMDs and (2) if so, what precisely was collected, based on items from questionnaires used and/or themes and subthemes identified from qualitative assessments. The two investigators then synthesized their findings in a schema of patient perceptions of interventions using BMDs.

**Results:**

A total of 58 RCTs including 10,071 participants were included in the review (the median number of randomized participants was 60, IQR 37-133). BMDs used in interventions were accelerometers/pedometers (n=35, 60%), electrochemical biosensors (eg, continuous glucose monitoring; n=18, 31%), or ecological momentary assessment devices (eg, carbon monoxide monitors for smoking cessation; n=5, 9%). Overall, 26 (45%) trials collected information on patient perceptions toward the intervention using BMDs and allowed the identification of 76 unique aspects of patient perceptions that could affect the uptake of these interventions (eg, relevance of the information provided, alarm burden, privacy and data handling, impact on health outcomes, independence, interference with daily life). Patient perceptions were unevenly collected in trials. For example, only 5% (n=3) of trials assessed how patients felt about privacy and data handling aspects of the intervention using BMDs.

**Conclusions:**

Our review showed that less than half of RCTs evaluating interventions using BMDs assessed patients’ perceptions toward interventions using BMDs. Trials that did assess perceptions often only assessed a fraction of them. This limits the extrapolation of the results of these RCTs to the real world. We thus provide a comprehensive schema of aspects of patient perceptions that may affect the uptake of interventions using BMDs and which should be considered in future trials.

**Trial Registration:**

PROSPERO CRD42018115522; https://tinyurl.com/y5h8fjgx

## Introduction

Biometric monitoring devices (BMDs) are wearable or environmental trackers and devices with embedded sensors that can remotely collect high-frequency objective data on patients’ physiological, biological, behavioral, and environmental contexts [1]. In recent years, there has been a surge of therapeutic interventions using BMDs to monitor patients’ health and treatment response to reactively adjust patients’ care “just in time” [1-7]. The development of these innovative interventions using BMDs has raised great interest from governments, payers, care providers, and patients given their potential to transform the delivery of care from intermittent clinical visits with clinicians to remote and continuous management of patients, at scale, in real time [2,7-10].

Despite promising results, the real-world effectiveness of interventions using BMDs depends on patients’ uptake, engagement, and adherence to these interventions [11]. For example, there is evidence of low patient engagement in the first large-scale implementations of digital monitoring strategies (eg, 90% incomplete follow-up for MyHeart Counts; 55% incomplete follow-up data for the Healthy Pregnancy Research Program) [12,13].

The literature on reasons explaining the poor uptake of these interventions, specifically on patients’ perceptions that can affect the uptake of interventions using BMDs is limited to the following: (1) small-sized pilot studies with short follow-ups [14-16], (2) surveys that explore stated preferences from patients [11,17], and (3) more rarely, objective assessment of patients’ perceptions toward these interventions in the clinical trials evaluating them (eg, via questionnaires). As a result, it is still unclear which specific patient perceptions should be measured in the clinical trials evaluating interventions using BMDs to inform their potential uptake. Such knowledge would strengthen inference about the potential external validity of results and benefit the planning of future trials.

In this study, we aimed to systematically review recent RCTs evaluating interventions using BMDs to understand whether and how patients’ perceptions toward these technologies were considered.

## Methods

We uploaded a prespecified protocol in November 2018 on PROSPERO (The International Prospective Register of Systematic Reviews; CRD42018115522). We followed standard procedures for systematic reviews and reported processes and results according to PRISMA (Preferred Reporting Items for Systematic Reviews and Meta-Analyses) [18].

### Data Sources and Searches

We systematically searched PubMed for eligible studies published in MEDLINE between January 1, 2017, and December 31, 2018. These eligibility dates were chosen to provide a sample of recent trials reflecting the current state of science on interventions using BMDs. The search equation had no language restrictions and was derived from the Cochrane Highly Sensitive Search Strategy with a filter for randomized controlled trials Medical Subject Heading (MeSH) terms, and free-text words pertaining to digital, mobile, and electronic health keywords identified during a pilot phase (Multimedia Appendix 1).

### Study Selection

We included published primary reports of RCTs in humans that assessed the efficacy of an intervention using BMDs (ie, interventions using wearables, trackers, or sensors/biosensors—for combined home and mobile use—that have the capability to collect and transmit data for the purposes of improving a patient’s health or preventing disease onset) [9,19,20]. When an intervention involved multiple components including some not related to BMDs, we focused on the component(s) involving BMDs. We excluded interventions utilizing telemedicine/telehealth (eg, videoconferencing), SMS text messages sent to mobile phones, and exclusively smartphone apps [21]. We excluded protocols, observational studies, and reviews. We also excluded publications evaluating interventions that were confined to a doctor’s office (eg, virtual reality headset intervention for the treatment of social anxiety disorder), and publications on interventions targeting clinicians rather than patients.

One investigator (AP) screened titles and abstracts for irrelevant publications. AP confirmed the eligibility of all screened-in studies based on the articles’ full-text and the reasons for not meeting eligibility.

### Data Extraction

One investigator (AP) used a standardized form to extract from the articles (and supplementary material and referenced sources if necessary) the general characteristics of trials (authors, title, journal, publication year, number of participants randomized, technology being assessed). When possible, we also reviewed the trial’s entry in a public clinical trial registry (eg, ClinicalTrials.gov) using information available in published articles. We assessed whether some outcomes measuring patients’ perceptions could be registered but not reported in published articles.

Two investigators (AP, MB) used a standardized form to independently extract data on how patients’ perceptions toward interventions using BMDs were assessed. These data included the following: (1) whether the trial collected information on patients’ perceptions toward the interventions using BMDs, (2) whether the information collected was a study outcome (primary or secondary), (3) how this information was measured (eg, using questionnaires, interviews, focus groups, or a combination of these), and (4) which patient perceptions were collected. This latter extraction was based on the review of all items from questionnaires used to assess patients’ perceptions toward interventions using BMDs, and/or themes and subthemes from qualitative assessments (ie, interviews and focus groups), if available. All items, themes and subthemes extracted were then compiled into a comprehensive list of patient perceptions toward the BMDs that were assessed in the included trials. Therefore, the list provided information on patient perceptions toward interventions using BMDs that may affect their uptake from both researchers’ (from the standardized questionnaires used in the RCTs) and patients’ perspectives (eg, from the qualitative assessments obtained in the RCTs).

### Data Synthesis and Analysis

#### General Characteristics of RCTs

We summarized the characteristics of included trials with frequencies (proportions) for categorical variables and medians and interquartile ranges (IQR) for continuous variables.

#### Schema of Patients’ Perceptions That Could Affect the Uptake of Interventions Using BMDs

Two investigators (AP and MB) independently organized the list of patient perceptions toward interventions using BMDs by critically examining the wording of the extracted content and context. First, they excluded general assessments (eg, whether the device was acceptable or helpful, in general) and restricted the list to specific patient perceptions toward interventions using BMDs that could affect the uptake of interventions. Second, they grouped similar patient perceptions (eg, “easy to use” and “I thought this system was easy to use” were grouped together as “easy to use”). Finally, they organized these perceptions into a schema of specific aspects of patient perceptions. Disagreements were collaboratively settled with a third investigator (VTT).

#### How RCTs and Validated Scales Cover the Schema of Patient Perceptions

We investigated how the trials included in this review covered the schema of patients’ perceptions toward interventions using BMDs by mapping the specific aspects of patients’ perceptions measured in each trial to the overarching categories and subcategories of the schema.

Similarly, we investigated how comprehensively the validated scales used in the included trials covered the schema by mapping specific aspects of patients’ perceptions from each validated questionnaire to the categories and subcategories of the schema.

Members of the public were not involved in the design of this systematic review or the interpretation of the results.

## Results

### General Characteristics of RCTs

**Figure 1:**
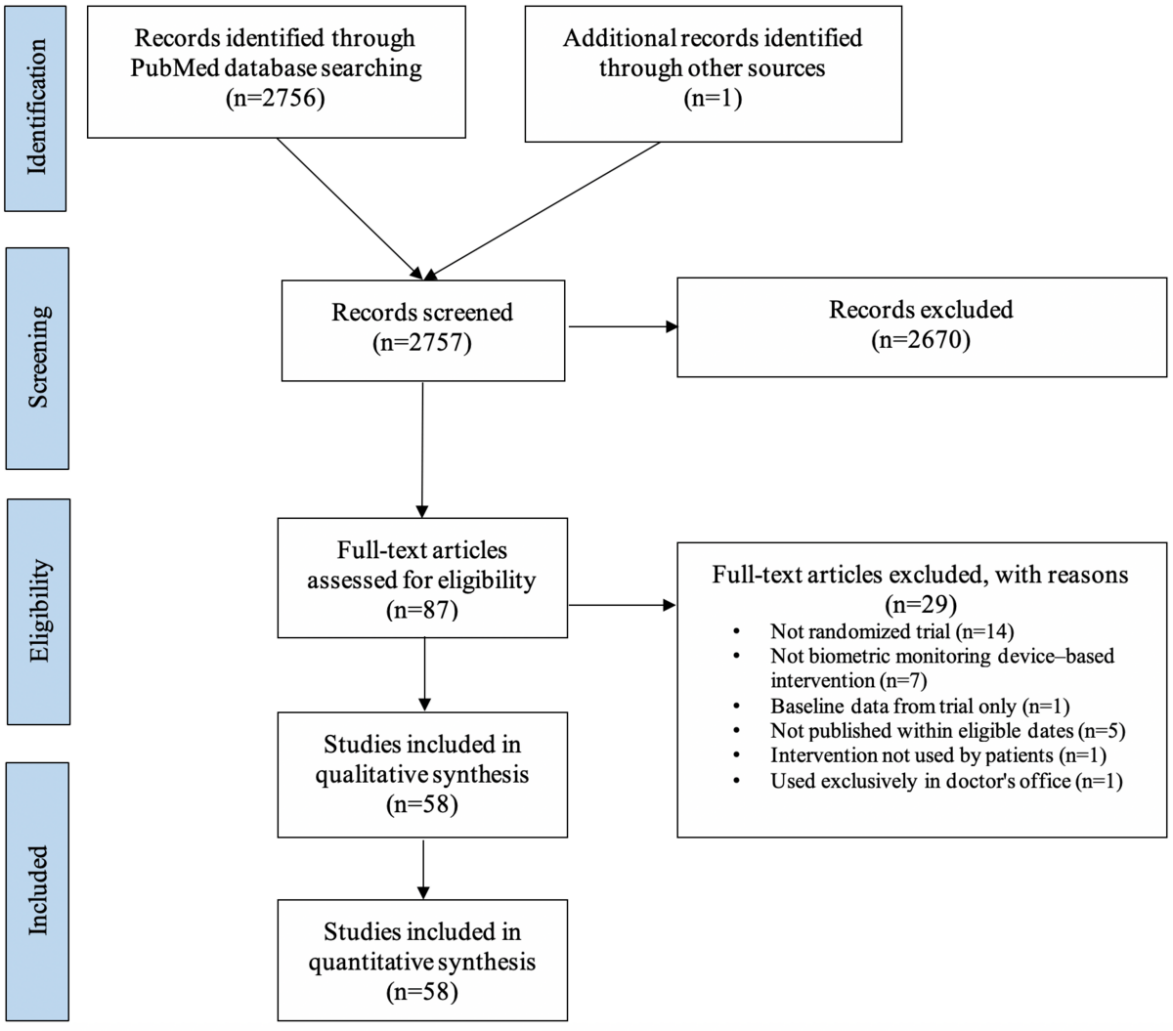
PRISMA flowchart. PRISMA: Preferred Reporting Items for Systematic Reviews and Meta-Analyses.

In total, 58 RCTs that randomized 10,071 participants were included in the review (Figure 1, Multimedia Appendix 2). RCTs randomized a median of 60 participants (IQR 37-133). Trials involved patients with diabetes (n=12, 21%), cancer (n=5, 9%), or healthy (or at-risk) primary patients (n=15, 26%). Trials were mostly funded by nonprofit sources (n=40, 69%); there were 5 (9%) trials that did not report their funding source. Most trials were single-center (n=54, 93%) and tested a commercialized technology (n=47, 81%).

BMDs used in interventions were mainly accelerometers/pedometers (eg, Fitbit; n=35, 60%), electrochemical biosensors (eg, continuous glucose monitoring devices; n=18, 31%), or ecological momentary assessment devices (n=5, 9%) that were either worn (eg, blood pressure monitor) or unworn (eg, carbon monoxide measurement monitors designed for smoking cessation; Table 1).

In total, 28 (48%) and 26 (45%) of the 58 included RCTs discussed and collected information on patients’ perceptions about the intervention using BMDs, respectively. Overall, 20 (34%) trials explicitly stated that the collected perceptions were trial outcomes. All 26 trials that collected perceptions reported how they were collected (eg, questionnaire, interview, focus group): 18 (31%) trials used a questionnaire, with 5 (9%) reporting that they used a validated instrument (Table 2).

**Table 1:** Characteristics of the 58 included trials (N=58)^a^.

Characteristic		Trials
Number of patients randomized, median (IQR)		60 (37-133)
**Type of biometric monitoring device^b^, n (%)**		
	Accelerometer/pedometer	35 (60)
	Electrochemical biosensor	18 (31)
	Ecological momentary assessment/attachable	5 (9)
**Therapeutic area, n (%)**		
	Diabetes	12 (21)
	Improving physical activity (primary prevention)	12 (21)
	Improving diet (primary prevention)	3 (5)
	Cardiovascular diseases (including stroke)	10 (17)
	Cancer	5 (9)
	Rheumatologic diseases	5 (9)
	Smoking/alcohol cessation	3 (5)
	Respiratory diseases	3 (5)
	Weight management	2 (3)
	Neurological diseases	2 (3)
	Gastrointestinal diseases	1 (2)
**Single or multicenter, n (%)**		
	Single center trial	54 (93)
	Multicenter trial	4 (7)
**Use of a commercial** **biometric monitoring device** **, n (%)**		
	Yes	47 (81)
	No	10 (17)
	Unknown	1 (2)
**Funding, n (%)**		
	Nonprofit (government, university, nonprofit nongovernmental organization)	40 (69)
	For-profit (pharmaceutical industries)	8 (14)
	Mixed	5 (9)
	Not reported	5 (9)

^a^Percentages may not equal 100% due to rounding.

^b^Many of these biometric monitoring devices were used in addition to a smartphone application.

**Table 2:** Collection, discussion, and reporting of patient perceptions toward biometric monitoring devices in the 58 included trials.

Collection, discussion, and reporting of patient perceptions		Studies, n (%)
Discussed at least one patient perception		28 (48)
**Collected at least one patient perception**		26 (45)
	With a questionnaire	18 (31)
	With face-to-face interviews	2 (3)
	With focus group	1 (2)
	By combining multiple collection modalities	5 (9)
Patient perception was reported as a trial outcome		20 (34)

### Schema of Patients’ Perceptions That Could Affect the Uptake of Interventions Using BMDs

Among the 26 trials that collected patients’ perceptions toward the intervention using BMDs, 23 (39%) evaluated specific patient perceptions of the intervention that could affect the uptake (ie, 3 collected only general satisfaction with or acceptability of the BMD).

**Figure 2:**
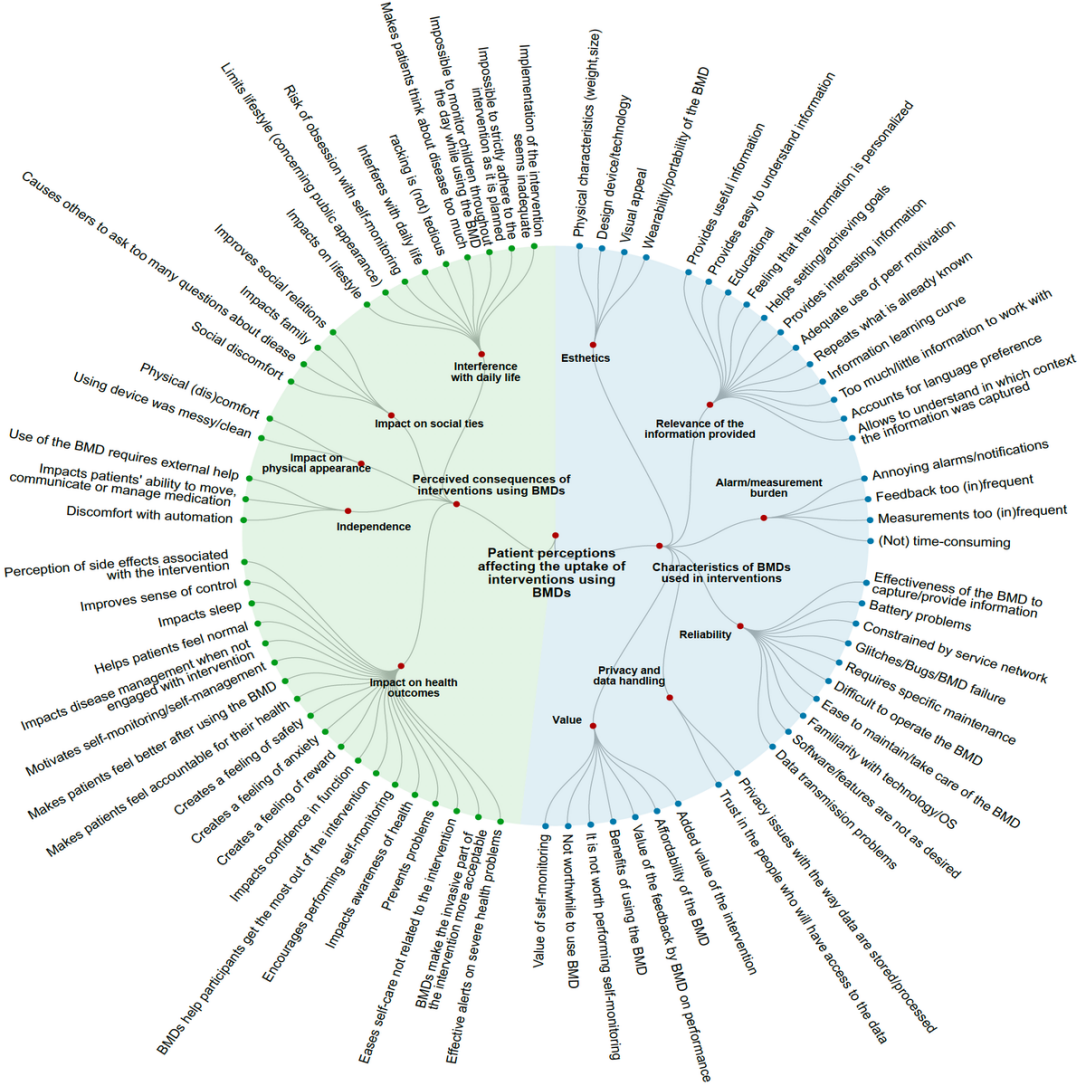
Schema of the 76 specific patient perceptions that could affect the uptake of interventions using BMDs. Specific perceptions are represented by blue nodes around the figure and organized in subcategories (outer red nodes) and major categories (inner red nodes dividing the circle into green and blue sections). BMD: biometric monitoring device.

We identified 76 unique specific aspects of patients’ perceptions toward interventions using BMDs that could affect their uptake. These aspects of perceptions were grouped into two overarching categories: (1) patient perceptions toward characteristics of BMDs used in interventions (n=39, 51%) and (2) perceived consequences of interventions using BMDs (n=37, 49%; Figure 2).

### Patients’ Perceptions Toward Characteristics of BMDs Used in Interventions

Patients’ perceptions toward the characteristics of BMDs were related to the following:

1. Esthetics, which describes the look and feel of the BMD. For instance, in one trial, patients were asked to assess whether the BMD was attractive and visually appealing. A total of 6 (10%) trials measured this patient perception [22].
2. Relevance of the information provided, which describes how well the patient feels he or she can interact with or use the information that the BMD delivers. For instance, in one trial, participants reported that potential further development of the BMD could include “more interesting content” on a web-based mobile service related to their use of a wrist-worn physical activity monitor [23]. A total of 15 (26%) trials measured this patient perception.
3. Alarm/measurement burden, which describes patients’ views about the BMDs’ features, such as alarm frequency or how frequently a measurement occurs. For instance, one trial asked patients to rate how much they agreed with the statement “Alarms too often for no good reason” [24]. A total of 6 (10%) trials measured this patient perception.
4. Reliability, which describes whether patients feel that the BMD used in the intervention can function properly (battery, connectivity, maintenance). For instance, one trial reported that “two participants discontinued using the Fitbit…because of battery problems” [25]. A total of 7 (12%) trials measured this patient perception.
5. Privacy and data handling, which describes how much patients feel that their privacy is protected and how accountable the people/organizations with whom their data is shared will use it for genuinely medical reasons. For instance, one trial asked patients how much they agreed with the statement “My privacy was protected when I used the system” [26]. A total of 3 (5%) trials measured this patient perception.
6. Value, which describes what patients can accept to forgo in terms of time or money for the intervention. For instance, one trial asked how much patients agreed with the statement “The effort of using this technology/method is worthwhile for me” [27]. A total of 10 (17%) trials measured this patient perception.

### Perceived Consequences of Interventions Using BMDs

Patients’ perceptions related to the potential consequences of the interventions using BMDs involved the following:

1. Perceived impact on health outcomes, which describes how the intervention may impact the patients’ health, disease, or response to treatment. For instance, one trial asked patients how much they agreed with the statement “Has helped to control diabetes better even when not wearing it” [24]. A total of 12 (21%) trials measured this patient perception.
2. Independence, which describes how the BMD may impact patients’ dependence on others or automation to conduct tasks. For instance, one trial asked participants how much they agreed with the statement “I felt that I needed someone's help to be able to use the system” [26]. A total of 9 (16%) trials measured this patient perception.
3. Perceived impact on their physical appearance, which describes patients’ views about how the BMD can impact their appearance or make them feel (physically). For instance, a questionnaire in one trial asked patients, “How physically uncomfortable was wearing the bracelet?” [28]. A total of 11 (19%) trials measured this patient perception.
4. Social ties, which describes how patients feel the intervention using the BMD makes them engage with other people and vice versa. For instance, one trial asked participants how much they agreed with the statement “Has caused more family arguments” [24]. A total of 6 (10%) trials measured this patient perception.
5. Interference, which describes how the intervention using the BMD interferes with daily life or alleviates daily stressors, and how patients feel about modifying their lifestyle to use the BMD. For instance, one trial asked patients how much they agreed with the statement “Causes too many hassles in daily life” [29]. A total of 12 (21%) trials measured this patient perception.

### How RCTs and Validated Scales Cover the Schema of Patient Perceptions

**Figure 3:**
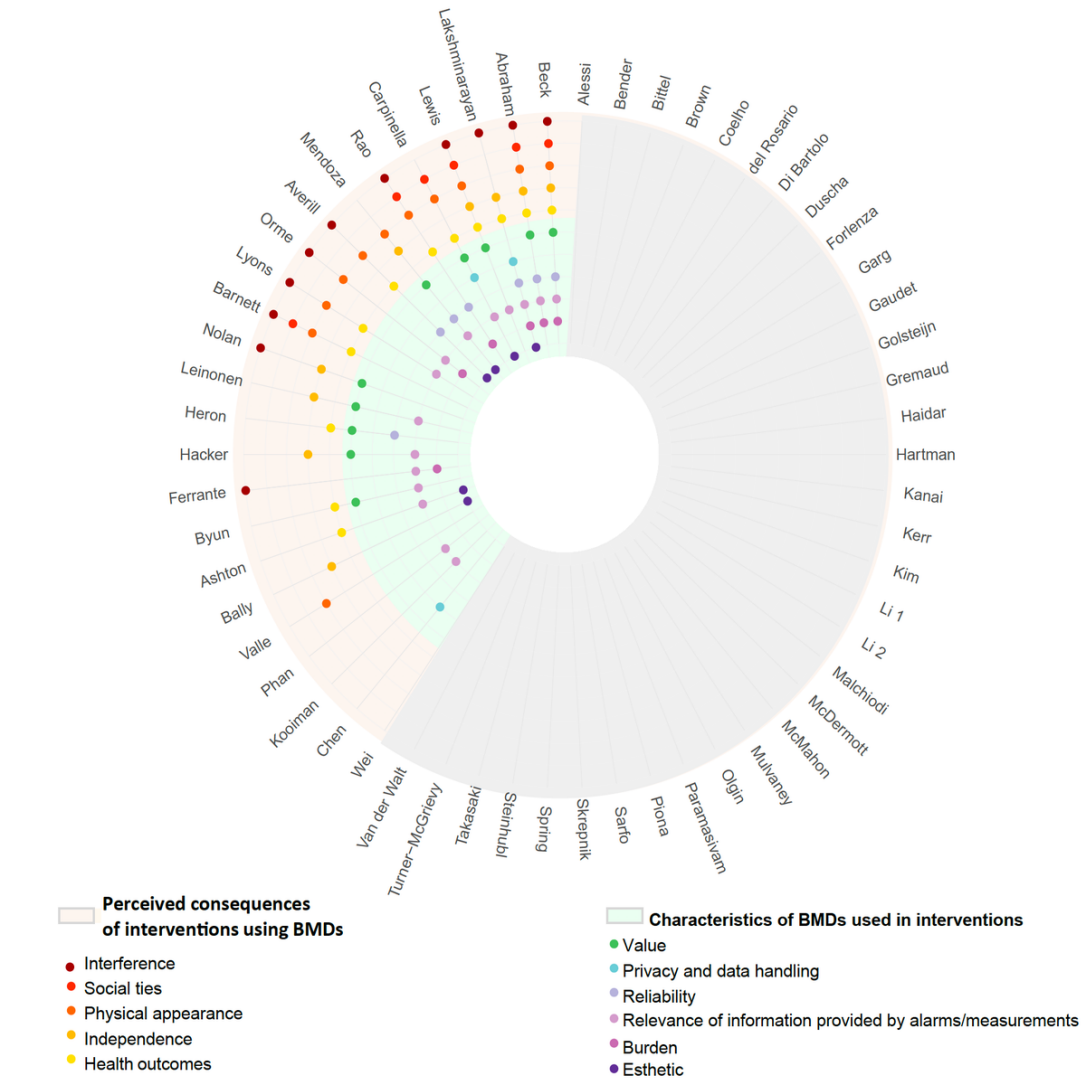
Patients’ perceptions toward interventions using BMDs collected in the included trials (n=58). All RCTs included in the current systematic review are shown around the figure by the first author’s last name. RCTs that collected at least one specific patient perception toward BMDs are shown in light green shading (category indicating patients’ perceptions toward characteristics of BMDs used in interventions) or beige shading (category indicating patients’ perceptions of consequences of interventions using BMDs). Gray shading corresponds to RCTs not collecting a specific patient perception toward the intervention using BMDs. Colored nodes in the interior of the figure correspond to subcategories of patient perceptions toward interventions using BMDs according to the schema in Figure 2. BMD: biometric monitoring device; RCT: randomized controlled trial.

Of the 23 trials that collected at least one specific aspect of a patient perception, 18 (78%) covered both perceptions toward characteristics of BMDs and perceptions of potential consequences of the intervention. Trials covered a median of 4 of the schema’s 11 subcategories (IQR 3-6, maximum 9). Furthermore, 8 of the trials covered 5 or more of the subcategories (Figure 3).

In the included trials, we identified four validated scales to measure patient perceptions toward the intervention using a BMD:

1. The 44-item Continuous Glucose Monitoring Satisfaction Questionnaire, used in two trials [24,29], covered 9/39 perceptions toward characteristics of BMDs and 17/37 perceptions of potential consequences of interventions using BMDs.
2. The 29-item Tele-healthcare Satisfaction Questionnaire used in one trial [27] covered 6/39 perceptions toward characteristics of BMDs and 5/37 perceptions of potential consequences of interventions using BMDs.
3. The 16-item Marshfield Usability Survey used in one trial [26] covered 4/39 perceptions toward characteristics of BMDs and 2/37 perceptions of potential consequences of interventions using BMDs.
4. The 17-item questionnaire adapted from Vandelanotte et al [30] used in one trial [31] covered 5/39 perceptions toward characteristics of BMDs and 3/37 perceptions of potential consequences of interventions using BMDs.

## Discussion

In this systematic review, we assessed how patients’ perceptions toward interventions using BMDs were assessed in recent RCTs. Our results highlight that less than half of trials collected patients’ perceptions toward the intervention. Among trials that did, most only partially covered the potential patient perceptions that could affect the uptake of interventions using BMDs. For example, only 5% of included trials assessed how patients felt with the privacy and data handling aspects of the intervention using BMDs. As a result, this creates an information gap regarding the potential uptake and implementation of these interventions [32,33].

Further, our work enabled the identification of a comprehensive list of 76 specific aspects of patients’ perceptions toward interventions using BMDs that could affect their uptake, coming from both investigators’ insights (through the analysis of the questionnaires used in the trials) and patients’ perspectives (through the inclusion of results from qualitative inquiries collected during trials). Our findings may help researchers developing new interventions using BMDs consider and address all aspects that could impact the uptake of their interventions.

To our knowledge, this is the first study to provide a comprehensive schema of patients’ perceptions toward interventions using BMDs. Our findings fit the empirical examples [34-37] of theoretical models about patient perspectives’ relationships with technology adoption [38,39] in that patients express views concerning ease of use, lack of privacy, enjoyment, motivation, and social influence. Our work is also more nuanced, emphasizing patients’ views about device affordability, reliability, relevance of information and content, value, and interference (with daily life), among many others.

Our first major result is that less than half of the trials in this review collected patient perceptions toward interventions using BMDs. These patients’ views are crucial to knowing whether the interventions would function in real-world settings and measuring them is the only way to get an insight into the potential uptake of these interventions in the real world [32,33]. In particular, we advocate against equating retention in trials with BMD adoption because retention is affected by the context of research.

Our second major result is that the patients’ perceptions toward interventions using BMDs collected in trials are numerous. Our results highlight that no scale used to measure patients’ perceptions toward interventions using BMDs offered a comprehensive assessment of the potential uptake of the interventions. Our schema of patient perceptions provides an empirical framework for helping guide implementation of the results of trials using BMDs, with the ultimate goal of wide-scale adoption in real-world settings. For example, it may serve the development of a new measurement tool for future trials.

Our findings complement the existing literature exploring the factors that may affect the uptake of BMDs and interventions using BMDs in health care, which was mainly composed of the following: (1) small-scale qualitative studies and theoretical models of technology adoption, (2) small-scale pilot studies testing the BMDs in controlled environments, and (3) surveys exploring stated preferences from patients. Individually, these studies did not capture the abundance and context of patients’ views toward BMDs and their adoption. For instance, theoretical models of technology adoption were not necessarily health care–specific. Pilot studies of interventions using BMDs often have short follow-up periods, and views expressed about BMDs may not be generalizable because of the limited sample size [14-16]. Qualitative studies or surveys explore stated preferences from patients [11,17] and often explore the general perceptions of people rather than their experience with specific BMDs in their own daily lives. Finally, there are some clinical trials that were included in our review in which patients’ perceptions toward BMDs were evaluated. However, unlike individual studies, this review organized the patients’ perceptions from all trials into a single schema. To our knowledge, our results present the most comprehensive assessment of patients’ perceptions toward BMDs that exists, which will help investigators and sponsors refine interventions to improve patients’ uptake and engagement.

Our study has some limitations. First, our inclusion criteria limited this review to RCTs (ie, preliminary observational pilot studies were excluded). However, we argue that these pilot studies evaluating new interventions using BMDs do not usually include many participants. Second, the schema we created is one of ostensibly multiple schemas that could have been created. Even though the systematically executed extraction would aid other investigators’ attempts to reproduce our findings, other investigators could create a different schema than ours, based on their experience. Third, we may have missed trials using BMDs by virtue of these devices being novel. Medical Subject Heading (MeSH) terms may not have been assigned yet or the assigned MeSH terms may not have included the ones from our search. Although our review was probably missing some trials, this would not have changed our main results that a large number of patient perceptions may affect the uptake of interventions using BMDs and that most trials did not adequately cover all of them. Fourth, we only searched one database as a trade-off between feasibility and potential impact on results. As our work is a methodological review describing the characteristics of RCTs evaluating interventions using BMDs, we do not need the same exhaustivity as a meta-analysis to evaluate a therapeutic intervention; thus, the omission of some studies published in journals not indexed in MEDLINE is unlikely to change the results. Fifth, screening of search results’ titles and abstracts was conducted by only one investigator (AP) instead of multiple assessors and could have resulted in the omission of some eligible trials.

A large number of patient perceptions can impact the uptake of a particular intervention using BMDs, help predict their real-world adoption, and guide the implementation of such interventions in routine clinical care. However, only a few of these perceptions are measured and only in fewer than half of clinical trials. Our review provides a simple schema of 11 important subcategories that comprehensively cover the factors that may affect the adoption of interventions using BMDs and could guide the development of future interventions. Future research should consider how intervention and BMD characteristics relate to perceptions toward interventions involving BMDs.

## Appendix

Multimedia Appendix 1 Search strategy.

Multimedia Appendix 2 References of included studies.

Multimedia Appendix 3 Characteristics of the 58 included trials by whether RCT reports collected general and specific information on patients’ perceptions about the intervention using BMDs.

## Abbreviations

BMD: biometric monitoring device
MeSH: Medical Subject Heading
RCT: randomized controlled trial

## References

[1] Arnerić SP, Cedarbaum J, Khozin S, Papapetropoulos S, Hill D, Ropacki M, Rhodes JA, Dacks PA, Hudson LD, Gordon MF, Kern VD, Romero K, Vradenburg G, Au R, Karlin DR, Facheris MF, Fitzer-Attas CJ, Vitolo OV, Wang J, Miller BM, Kaye JA (2017). Biometric monitoring devices for assessing end points in clinical trials: developing an ecosystem. Nat Rev Drug Discov.

[2] Elenko E, Underwood L, Zohar D (2015). Defining digital medicine. Nat Biotechnol.

[3] Green EM, van Mourik R, Wolfus C, Heitner SB, Dur O, Semigran MJ (2019). Machine learning detection of obstructive hypertrophic cardiomyopathy using a wearable biosensor. NPJ Digit Med.

[4] Carreiro S, Fang H, Zhang J, Wittbold K, Weng S, Mullins R, Smelson D, Boyer EW (2015). iMStrong: Deployment of a Biosensor System to Detect Cocaine Use. J Med Syst.

[5] Liao Y, Thompson C, Peterson S, Mandrola J, Beg MS (2019). The Future of Wearable Technologies and Remote Monitoring in Health Care. American Society of Clinical Oncology Educational Book.

[6] Singh R, Lewis B, Chapman B, Carreiro S, Venkatasubramanian K (2019). A Machine Learning-based Approach for Collaborative Non-Adherence Detection during Opioid Abuse Surveillance using a Wearable Biosensor. Biomed Eng Syst Technol Int Jt Conf BIOSTEC Revis Sel Pap.

[7] Perez MV, Mahaffey KW, Hedlin H, Rumsfeld JS, Garcia A, Ferris T, Balasubramanian V, Russo AM, Rajmane A, Cheung L, Hung G, Lee J, Kowey P, Talati N, Nag D, Gummidipundi SE, Beatty A, Hills MT, Desai S, Granger CB, Desai M, Turakhia MP (2019). Large-Scale Assessment of a Smartwatch to Identify Atrial Fibrillation. N Engl J Med.

[8] Ajana B (2017). Digital health and the biopolitics of the Quantified Self. Digit Health.

[9] Rich E, Miah A (2016). Mobile, wearable and ingestible health technologies: towards a critical research agenda. Health Sociology Review.

[10] Ruckenstein M, Schüll ND (2017). The Datafication of Health. Annu Rev Anthropol.

[11] Tran V, Riveros C, Ravaud P (2019). Patients' views of wearable devices and AI in healthcare: findings from the ComPaRe e-cohort. NPJ Digit Med.

[12] McConnell MV, Shcherbina A, Pavlovic A, Homburger JR, Goldfeder RL, Waggot D, Cho MK, Rosenberger ME, Haskell WL, Myers J, Champagne MA, Mignot E, Landray M, Tarassenko L, Harrington RA, Yeung AC, Ashley EA (2017). Feasibility of Obtaining Measures of Lifestyle From a Smartphone App: The MyHeart Counts Cardiovascular Health Study. JAMA Cardiol.

[13] Radin J, Steinhubl S, Su A, Bhargava H, Greenberg B, Bot B, Doerr M, Topol EJ (2018). The Healthy Pregnancy Research Program: transforming pregnancy research through a ResearchKit app. NPJ Digit Med.

[14] Manini TM, Mendoza T, Battula M, Davoudi A, Kheirkhahan M, Young ME, Weber E, Fillingim RB, Rashidi P (2019). Perception of Older Adults Toward Smartwatch Technology for Assessing Pain and Related Patient-Reported Outcomes: Pilot Study. JMIR Mhealth Uhealth.

[15] Grando MA, Bayuk M, Karway G, Corrette K, Groat D, Cook CB, Thompson B (2019). Patient Perception and Satisfaction With Insulin Pump System: Pilot User Experience Survey. J Diabetes Sci Technol.

[16] Kropff J, DeJong J, Del Favero S, Place J, Messori M, Coestier B, Farret A, Boscari F, Galasso S, Avogaro A, Bruttomesso D, Cobelli C, Renard E, Magni L, DeVries JH, AP@home consortium (2017). Psychological outcomes of evening and night closed-loop insulin delivery under free living conditions in people with Type 1 diabetes: a 2-month randomized crossover trial. Diabet Med.

[17] Mosconi P, Radrezza S, Lettieri E, Santoro E (2019). Use of Health Apps and Wearable Devices: Survey Among Italian Associations for Patient Advocacy. JMIR mHealth uHealth.

[18] Moher D, Liberati A, Tetzlaff J, Altman DG, PRISMA Group (2009). Preferred reporting items for systematic reviews and meta-analyses: the PRISMA statement. PLoS Med.

[19] Zhang D, Liu Q (2016). Biosensors and bioelectronics on smartphone for portable biochemical detection. Biosens Bioelectron.

[20] Sezgin E, Yildirim S, Özkan-Yildirim S, Sumuer E (2018). Current and Emerging mHealth Technologies: Adoption, Implementation, and Use.

[21] Chen CE, Harrington RA, Desai SA, Mahaffey KW, Turakhia MP (2019). Characteristics of Digital Health Studies Registered in ClinicalTrials.gov. JAMA Intern Med.

[22] Ashton LM, Morgan PJ, Hutchesson MJ, Rollo ME, Collins CE (2017). Feasibility and preliminary efficacy of the 'HEYMAN' healthy lifestyle program for young men: a pilot randomised controlled trial. Nutr J.

[23] Leinonen A, Pyky R, Ahola R, Kangas M, Siirtola P, Luoto T, Enwald H, Ikäheimo TM, Röning J, Keinänen-Kiukaanniemi S, Mäntysaari M, Korpelainen R, Jämsä T (2017). Feasibility of Gamified Mobile Service Aimed at Physical Activation in Young Men: Population-Based Randomized Controlled Study (MOPO). JMIR Mhealth Uhealth.

[24] Beck RW, Riddlesworth T, Ruedy K, Ahmann A, Bergenstal R, Haller S, Kollman C, Kruger D, McGill JB, Polonsky W, Toschi E, Wolpert H, Price D, DIAMOND Study Group (2017). Effect of Continuous Glucose Monitoring on Glycemic Control in Adults With Type 1 Diabetes Using Insulin Injections: The DIAMOND Randomized Clinical Trial. JAMA.

[25] Heron N, Kee F, Mant J, Reilly PM, Cupples M, Tully M, Donnelly M (2017). Stroke Prevention Rehabilitation Intervention Trial of Exercise (SPRITE) - a randomised feasibility study. BMC Cardiovasc Disord.

[26] Lakshminarayan K, Westberg S, Northuis C, Fuller CC, Ikramuddin F, Ezzeddine M, Scherber J, Speedie S (2018). A mHealth-based care model for improving hypertension control in stroke survivors: Pilot RCT. Contemp Clin Trials.

[27] Carpinella I, Cattaneo D, Bonora G, Bowman T, Martina L, Montesano A, Ferrarin M (2017). Wearable Sensor-Based Biofeedback Training for Balance and Gait in Parkinson Disease: A Pilot Randomized Controlled Trial. Arch Phys Med Rehabil.

[28] Barnett NP, Celio MA, Tidey JW, Murphy JG, Colby SM, Swift RM (2017). A preliminary randomized controlled trial of contingency management for alcohol use reduction using a transdermal alcohol sensor. Addiction.

[29] Abraham MB, Nicholas JA, Smith GJ, Fairchild JM, King BR, Ambler GR, Cameron FJ, Davis EA, Jones TW, PLGM Study Group (2018). Reduction in Hypoglycemia With the Predictive Low-Glucose Management System: A Long-term Randomized Controlled Trial in Adolescents With Type 1 Diabetes. Diabetes Care.

[30] Vandelanotte C, De Bourdeaudhuij I (2003). Acceptability and feasibility of a computer-tailored physical activity intervention using stages of change: project FAITH. Health Educ Res.

[31] Lyons EJ, Swartz MC, Lewis ZH, Martinez E, Jennings K (2017). Feasibility and Acceptability of a Wearable Technology Physical Activity Intervention With Telephone Counseling for Mid-Aged and Older Adults: A Randomized Controlled Pilot Trial. JMIR mHealth uHealth.

[32] Barnard KD, Pinsker JE, Oliver N, Astle A, Dassau E, Kerr D (2015). Future artificial pancreas technology for type 1 diabetes: what do users want?. Diabetes Technol Ther.

[33] Simblett S, Greer B, Matcham F, Curtis H, Polhemus A, Ferrão J, Gamble P, Wykes T (2018). Barriers to and Facilitators of Engagement With Remote Measurement Technology for Managing Health: Systematic Review and Content Analysis of Findings. J Med Internet Res.

[34] Gao Y, Li H, Luo Y (2015). An empirical study of wearable technology acceptance in healthcare. Industr Mngmnt & Data Systems.

[35] Li H, Wu J, Gao Y, Shi Y (2016). Examining individuals' adoption of healthcare wearable devices: An empirical study from privacy calculus perspective. Int J Med Inform.

[36] Lunney A, Cunningham NR, Eastin MS (2016). Wearable fitness technology: A structural investigation into acceptance and perceived fitness outcomes. Computers in Human Behavior.

[37] Mackert M, Mabry-Flynn A, Champlin S, Donovan EE, Pounders K (2016). Health Literacy and Health Information Technology Adoption: The Potential for a New Digital Divide. J Med Internet Res.

[38] Rahimi B, Nadri H, Lotfnezhad Afshar H, Timpka T (2018). A Systematic Review of the Technology Acceptance Model in Health Informatics. Appl Clin Inform.

[39] Holden RJ, Karsh B (2010). The technology acceptance model: its past and its future in health care. J Biomed Inform.

