# Supplementary material for: Identification of Patient Perceptions That Can Affect the Uptake of Interventions Using Biometric Monitoring Devices: Systematic Review of Randomized Controlled Trials
Source: J Med Internet Res. 2020 Sep 11;22(9):e18986. doi: 10.2196/18986 (PMC7519434; doi:10.2196/18986)
Supplement: Multimedia Appendix 1 [file jmir_v22i9e18986_app1.docx]

**Additional File 1: Search strategy**

1. randomized controlled trial [tiab] OR controlled clinical trial [tiab] OR randomized [tiab] OR placebo [tiab] OR drug therapy [sh] OR randomly [tiab] OR trial [tiab] OR groups [tiab]
2. animals[mh] NOT humans[mh]
3. #1 NOT #2
4. (Accelerometry/instrumentation*[MeSH]) OR (Accelerometry/methods*[MeSH]) OR (Biofeedback, Psychology/instrumentation*[MeSH]) OR (Biosensing Techniques/instrumentation*[MeSH]) OR (Biosensing Techniques/methods[MeSH]) OR (Drug Monitoring/instrumentation[MeSH]) OR (Ecological Momentary Assessment*[MeSH]) OR (Mobile Applications[MeSH]) OR (Monitoring, Ambulatory/instrumentation*[MeSH]) OR (Monitoring, Physiologic/instrumentation*[MeSH]) OR (Psychotherapy/instrumentation[MeSH]) OR (Reminder Systems/instrumentation*[MeSH]) OR (Smartphone[MeSH]) OR (Wearable Electronic Devices*[MeSH])) OR “mhealth” OR “ehealth” OR “mobile technology” OR “mobile health”)
5. #3 AND #4
6. Restrict publication dates to January 1, 2017 to December 31, 2018
