# Supplementary material for: Identification of Patient Perceptions That Can Affect the Uptake of Interventions Using Biometric Monitoring Devices: Systematic Review of Randomized Controlled Trials
Source: J Med Internet Res. 2020 Sep 11;22(9):e18986. doi: 10.2196/18986 (PMC7519434; doi:10.2196/18986)
Supplement: Multimedia Appendix 2 [file jmir_v22i9e18986_app2.docx]

**Additional File 2: References of included studies**

1. Abraham MB, Nicholas JA, Smith GJ, Fairchild JM, King BR, Ambler GR, et al. Reduction in Hypoglycemia With the Predictive Low-Glucose Management System: A Long-term Randomized Controlled Trial in Adolescents With Type 1 Diabetes. Diabetes Care. 2018;41(2):303-10.

2. Alessi SM, Rash CJ, Petry NM. A Randomized Trial of Adjunct mHealth Abstinence Reinforcement With Transdermal Nicotine and Counseling for Smoking Cessation. Nicotine Tob Res;19(3):290-8.

3. Ashton LM, Morgan PJ, Hutchesson MJ, Rollo ME, Collins CE. Feasibility and preliminary efficacy of the 'HEYMAN' healthy lifestyle program for young men: a pilot randomised controlled trial. Nutr J;16(1):2.

4. Averill F, Brown TG, Robertson RD, Tchomgang A, Berbiche D, Nadeau L, et al. Transdermal alcohol monitoring combined with contingency management for driving while impaired offenders: A pilot randomized controlled study. Traffic Inj Prev. 2018;19(5):455-61.

5. Bally L, Thabit H, Hartnell S, Andereggen E, Ruan Y, Wilinska ME, et al. Closed-Loop Insulin Delivery for Glycemic Control in Noncritical Care. New England Journal of Medicine. 2018;379(6):547-56.

6. Barnett NP, Celio MA, Tidey JW, Murphy JG, Colby SM, Swift RM. A preliminary randomized controlled trial of contingency management for alcohol use reduction using a transdermal alcohol sensor. Addiction;112(6):1025-35.

7. Beck RW, Riddlesworth T, Ruedy K, Ahmann A, Bergenstal R, Haller S, et al. Effect of Continuous Glucose Monitoring on Glycemic Control in Adults With Type 1 Diabetes Using Insulin Injections: The DIAMOND Randomized Clinical Trial. Jama;317(4):371-8.

8. Bender MS, Cooper BA, Flowers E, Ma R, Arai S. Filipinos Fit and Trim - A feasible and efficacious DPP-based intervention trial. Contemp Clin Trials Commun. 2018;12:76-84.

9. Bittel DC, Bittel AJ, Williams C, Elazzazi A. Improving Exercise Performance with an Accelerometer-Based Smartphone App: A Randomized Controlled Trial. Am J Phys Med Rehabil;96(5):307-14.

10. Brown SA, Breton MD, Anderson SM, Kollar L, Keith-Hynes P, Levy CJ, et al. Overnight Closed-Loop Control Improves Glycemic Control in a Multicenter Study of Adults With Type 1 Diabetes. J Clin Endocrinol Metab;102(10):3674-82.

11. Byun W, Lau EY, Brusseau TA. Feasibility and Effectiveness of a Wearable Technology-Based Physical Activity Intervention in Preschoolers: A Pilot Study. Int J Environ Res Public Health. 2018;15(9).

12. Carpinella I, Cattaneo D, Bonora G, Bowman T, Martina L, Montesano A, et al. Wearable Sensor-Based Biofeedback Training for Balance and Gait in Parkinson Disease: A Pilot Randomized Controlled Trial. Arch Phys Med Rehabil;98(4):622-30.e3.

13. Chen JL, Guedes CM, Cooper BA, Lung AE. Short-Term Efficacy of an Innovative Mobile Phone Technology-Based Intervention for Weight Management for Overweight and Obese Adolescents: Pilot Study. Interact J Med Res;6(2):e12.

14. Coelho CM, Reboredo MM, Valle FM, Malaguti C, Campos LA, Nascimento LM, et al. Effects of an unsupervised pedometer-based physical activity program on daily steps of adults with moderate to severe asthma: a randomized controlled trial. J Sports Sci;36(10):1186-93.

15. del Rosario MB, Lovell NH, Fildes J, Holgate K, Yu J, Ferry C, et al. Evaluation of an mHealth-Based Adjunct to Outpatient Cardiac Rehabilitation. IEEE J Biomed Health Inform. 2018;22(6):1938-48.

16. Di Bartolo P, Nicolucci A, Cherubini V, Iafusco D, Scardapane M, Rossi MC. Young patients with type 1 diabetes poorly controlled and poorly compliant with self-monitoring of blood glucose: can technology help? Results of the i-NewTrend randomized clinical trial. Acta Diabetol;54(4):393-402.

17. Duscha BD, Piner LW, Patel MP, Crawford LE, Jones WS, Patel MR, et al. Effects of a 12-Week mHealth Program on FunctionalCapacity and Physical Activity in Patients With PeripheralArtery Disease. Am J Cardiol. 2018;122(5):879-84.

18. Ferrante JM, Devine KA, Bator A, Rodgers A, Ohman-Strickland PA, Bandera EV, et al. Feasibility and potential efficacy of commercial mHealth/eHealth tools for weight loss in African American breast cancer survivors: pilot randomized controlled trial. Transl Behav Med. 2018.

19. Forlenza GP, Deshp, e S, Ly TT, Howsmon DP, Cameron F, et al. Application of Zone Model Predictive Control Artificial Pancreas During Extended Use of Infusion Set and Sensor: A Randomized Crossover-Controlled Home-Use Trial. Diabetes Care;40(8):1096-102.

20. Garg SK, Shah VN, Akturk HK, Beatson C, Snell-Bergeon JK. Role of Mobile Technology to Improve Diabetes Care in Adults with Type 1 Diabetes: The Remote-T1D Study iBGStar((R)) in Type 1 Diabetes Management. Diabetes Ther;8(4):811-9.

21. Gaudet J, Gallant F, Belanger M. A Bit of Fit: Minimalist Intervention in Adolescents Based on a Physical Activity Tracker. JMIR Mhealth Uhealth;5(7):e92.

22. Golsteijn RHJ, Bolman C, Volders E, Peels DA, de Vries H, Lechner L. Short-term efficacy of a computer-tailored physical activity intervention for prostate and colorectal cancer patients and survivors: a randomized controlled trial. Int J Behav Nutr Phys Act. 2018;15(1):106.

23. Gremaud AL, Carr LJ, Simmering JE, Evans NJ, Cremer JF, Segre AM, et al. Gamifying Accelerometer Use Increases Physical Activity Levels of Sedentary Office Workers. J Am Heart Assoc. 2018;7(13).

24. Hacker E, Horsham C, Vagenas D, Jones L, Lowe J, Janda M. A Mobile Technology Intervention With Ultraviolet Radiation Dosimeters and Smartphone Apps for Skin Cancer Prevention in Young Adults: Randomized Controlled Trial. JMIR Mhealth Uhealth. 2018;6(11):e199.

25. Haidar A, Messier V, Legault L, Ladouceur M, Rabasa-Lhoret R. Outpatient 60-hour day-and-night glucose control with dual-hormone artificial pancreas, single-hormone artificial pancreas, or sensor-augmented pump therapy in adults with type 1 diabetes: An open-label, randomised, crossover, controlled trial. Diabetes Obes Metab;19(5):713-20.

26. Hartman SJ, Nelson SH, Weiner LS. Patterns of Fitbit Use and Activity Levels Throughout a Physical Activity Intervention: Exploratory Analysis from a Randomized Controlled Trial. JMIR Mhealth Uhealth;6(2):e29.

27. Heron N, Kee F, Mant J, Reilly PM, Cupples M, Tully M, et al. Stroke Prevention Rehabilitation Intervention Trial of Exercise (SPRITE) - a randomised feasibility study. BMC Cardiovasc Disord;17(1):290.

28. Kanai M, Izawa KP, Kobayashi M, Onishi A, Kubo H, Nozoe M, et al. Effect of accelerometer-based feedback on physical activity in hospitalized patients with ischemic stroke: a randomized controlled trial. Clin Rehabil. 2018;32(8):1047-56.

29. Kerr A, Dawson J, Robertson C, Rowe P, Quinn TJ. Sit to stand activity during stroke rehabilitation. Top Stroke Rehabil;24(8):562-6.

30. Kim Y, Lumpkin A, Lochbaum M, Stegemeier S, Kitten K. Promoting physical activity using a wearable activity tracker in college students: A cluster randomized controlled trial. J Sports Sci. 2018;36(16):1889-96.

31. Kooiman TJM, de Groot M, Hoogenberg K, Krijnen WP, van der Schans CP, Kooy A. Self-tracking of Physical Activity in People With Type 2 Diabetes: A Randomized Controlled Trial. Comput Inform Nurs. 2018;36(7):340-9.

32. Lakshminarayan K, Westberg S, Northuis C, Fuller CC, Ikramuddin F, Ezzeddine M, et al. A mHealth-based care model for improving hypertension control in stroke survivors: Pilot RCT. Contemp Clin Trials. 2018;70:24-34.

33. Leinonen AM, Pyky R, Ahola R, Kangas M, Siirtola P, Luoto T, et al. Feasibility of Gamified Mobile Service Aimed at Physical Activation in Young Men: Population-Based Randomized Controlled Study (MOPO). JMIR Mhealth Uhealth;5(10):e146.

34. Lewis ZH, Ottenbacher KJ, Fisher SR, Jennings K, Brown AF, Swartz MC, et al. The feasibility and RE-AIM evaluation of the TAME health pilot study. Int J Behav Nutr Phys Act;14(1):106.

35. Li LC, Sayre EC, Xie H, Clayton C, Feehan LM. A Community-Based Physical Activity Counselling Program for People With Knee Osteoarthritis: Feasibility and Preliminary Efficacy of the Track-OA Study. JMIR Mhealth Uhealth;5(6):e86.

36. Li LC, Sayre EC, Xie H, Falck RS, Best JR, Liu-Ambrose T, et al. Efficacy of a Community-Based Technology-Enabled Physical Activity Counseling Program for People With Knee Osteoarthritis: Proof-of-Concept Study. J Med Internet Res;20(4):e159.

37. Lyons EJ, Swartz MC, Lewis ZH, Martinez E, Jennings K. Feasibility and Acceptability of a Wearable Technology Physical Activity Intervention With Telephone Counseling for Mid-Aged and Older Adults: A Randomized Controlled Pilot Trial. JMIR Mhealth Uhealth;5(3):e28.

38. Malchiodi Albedi G, Corna S, Aspesi V, Clerici D, Parisio C, Seitanidis J, et al. Effects of nanotechnology-based devices on postural control in healthy subjects. J Sports Med Phys Fitness. 2018;58(10):1418-22.

39. McDermott MM, Spring B, Berger JS, Treat-Jacobson D, Conte MS, Creager MA, et al. Effect of a Home-Based Exercise Intervention of Wearable Technology and Telephone Coaching on Walking Performance in Peripheral Artery Disease: The HONOR Randomized Clinical Trial. Jama;319(16):1665-76.

40. McMahon SK, Lewis B, Oakes JM, Wyman JF, Guan W, Rothman AJ. Assessing the Effects of Interpersonal and Intrapersonal Behavior Change Strategies on Physical Activity in Older Adults: a Factorial Experiment. Ann Behav Med;51(3):376-90.

41. Mendoza JA, Baker KS, Moreno MA, Whitlock K, Abbey-Lambertz M, Waite A, et al. A Fitbit and Facebook mHealth intervention for promoting physical activity among adolescent and young adult childhood cancer survivors: A pilot study. Pediatr Blood Cancer;64(12).

42. Mulvaney SA, Vaala S, Hood KK, Lybarger C, Carroll R, Williams L, et al. Mobile Momentary Assessment and Biobehavioral Feedback for Adolescents with Type 1 Diabetes: Feasibility and Engagement Patterns. Diabetes Technol Ther;20(7):465-74.

43. Nolan CM, Maddocks M, Canavan JL, Jones SE, Delogu V, Kaliaraju D, et al. Pedometer Step Count Targets during Pulmonary Rehabilitation in Chronic Obstructive Pulmonary Disease. A Randomized Controlled Trial. Am J Respir Crit Care Med;195(10):1344-52.

44. Olgin JE, Pletcher MJ, Vittinghoff E, Wranicz J, Malik R, Morin DP, et al. Wearable Cardioverter-Defibrillator after Myocardial Infarction. N Engl J Med. 2018;379(13):1205-15.

45. Orme MW, Weedon AE, Saukko PM, Esliger DW, Morgan MD, Steiner MC, et al. Findings of the Chronic Obstructive Pulmonary Disease-Sitting and Exacerbations Trial (COPD-SEAT) in Reducing Sedentary Time Using Wearable and Mobile Technologies With Educational Support: Randomized Controlled Feasibility Trial. JMIR Mhealth Uhealth;6(4):e84.

46. Paramasivam SS, Chinna K, Singh AKK, Ratnasingam J, Ibrahim L, Lim LL, et al. Continuous glucose monitoring results in lower HbA1c in Malaysian women with insulin-treated gestational diabetes: a randomized controlled trial. Diabet Med. 2018;35(8):1118-29.

47. Phan TT, Barnini N, Xie S, Martinez A, Falini L, Abatemarco A, et al. Feasibility of Using a Commercial Fitness Tracker as an Adjunct to Family-Based Weight Management Treatment: Pilot Randomized Trial. JMIR Mhealth Uhealth. 2018;6(11):e10523.

48. Piona C, Dovc K, Mutlu GY, Grad K, Gregorc P, Battelino T, et al. Non-adjunctive flash glucose monitoring system use during summer-camp in children with type 1 diabetes: The free-summer study. Pediatr Diabetes. 2018;19(7):1285-93.

49. Rao SSC, Valestin JA, Xiang X, Hamdy S, Bradley CS, Zimmerman MB. Home-based versus office-based biofeedback therapy for constipation with dyssynergic defecation: a randomised controlled trial. Lancet Gastroenterol Hepatol. 2018;3(11):768-77.

50. Sarfo F, Treiber F, Gebregziabher M, Adamu S, Patel S, Nichols M, et al. PINGS (Phone-Based Intervention Under Nurse Guidance After Stroke): Interim Results of a Pilot Randomized Controlled Trial. Stroke;49(1):236-9.

51. Skrepnik N, Spitzer A, Altman R, Hoekstra J, Stewart J, Toselli R. Assessing the Impact of a Novel Smartphone Application Compared With Standard Follow-Up on Mobility of Patients With Knee Osteoarthritis Following Treatment With Hylan G-F 20: A Randomized Controlled Trial. JMIR Mhealth Uhealth;5(5):e64.

52. Spring B, Pellegrini C, McFadden HG, Pfammatter AF, Stump TK, Siddique J, et al. Multicomponent mHealth Intervention for Large, Sustained Change in Multiple Diet and Activity Risk Behaviors: The Make Better Choices 2 Randomized Controlled Trial. J Med Internet Res;20(6):e10528.

53. Steinhubl SR, Waalen J, Edwards AM, Ariniello LM, Mehta RR, Ebner GS, et al. Effect of a Home-Based Wearable Continuous ECG Monitoring Patch on Detection of Undiagnosed Atrial Fibrillation: The mSToPS Randomized Clinical Trial. Jama. 2018;320(2):146-55.

54. Takasaki H, Aoki S, May S. No increase in 6-week treatment effect of Mechanical Diagnosis and Therapy with the use of the LUMOback in people with non-acute non-specific low back pain and a directional preference of extension: a pilot randomized controlled trial. Physiotherapy. 2018;104(3):347-53.

55. Turner-McGrievy GM, Wilcox S, Boutte A, Hutto BE, Singletary C, Muth ER, et al. The Dietary Intervention to Enhance Tracking with Mobile Devices (DIET Mobile) Study: A 6-Month Randomized Weight Loss Trial. Obesity (Silver Spring);25(8):1336-42.

56. Valle CG, Deal AM, Tate DF. Preventing weight gain in African American breast cancer survivors using smart scales and activity trackers: a randomized controlled pilot study. J Cancer Surviv;11(1):133-48.

57. Van der Walt N, Salmon LJ, Gooden B, Lyons MC, O'Sullivan M, Martina K, et al. Feedback From Activity Trackers Improves Daily Step Count After Knee and Hip Arthroplasty: A Randomized Controlled Trial. J Arthroplasty. 2018;33(11):3422-8.

58. Wei TY, Chang DW, Liu YD, Liu CW, Young CP, Liang SF, et al. Portable wireless neurofeedback system of EEG alpha rhythm enhances memory. Biomed Eng Online;16(1):128.
