# Supplementary material for: Identification of Patient Perceptions That Can Affect the Uptake of Interventions Using Biometric Monitoring Devices: Systematic Review of Randomized Controlled Trials
Source: J Med Internet Res. 2020 Sep 11;22(9):e18986. doi: 10.2196/18986 (PMC7519434; doi:10.2196/18986)
Supplement: Multimedia Appendix 3 [file jmir_v22i9e18986_app3.docx]

**Table S1. Characteristics of the 58 included trials by whether RCT reports collected general and specific information on patients’ perceptions about the intervention using BMDs.**

|  | **RCTs collected general and specific patient perceptions about the intervention using BMDs**  **(N=26)** | **RCTs did *not* collect general or specific patient perceptions about the intervention using BMDs**  **(N=32)** |
| --- | --- | --- |
| **Characteristic** | **N (%**^2^**)** | |
| **Number of patients randomized - median (IQR)** | 58 (36-117) | 66 (40-133) |
| **Type of biometric monitoring device^1^** |  |  |
| Accelerometer/pedometer | 17 (65) | 18 (56) |
| Electrochemical biosensor | 7 (27) | 11 (34) |
| Ecological momentary assessment/attachable | 2 (8) | 3 (9) |
| **Therapeutic area** |  |  |
| Diabetes | 5 (19) | 7 (22) |
| Improving physical activity (primary prevention) | 5 (19) | 7 (22) |
| Improving diet (primary prevention) | 1 (4) | 2 (6) |
| Cardiovascular diseases (incl. stroke) | 2 (8) | 8 (25) |
| Cancer | 4 (15) | 1 (3) |
| Rheumatologic diseases | 1 (4) | 4 (13) |
| Smoking/alcohol cessation | 2 (8) | 1 (3) |
| Respiratory diseases | 2 (8) | 1 (3) |
| Weight management | 2 (8) | 0 (0) |
| Neurological diseases | 1 (4) | 1 (3) |
| Gastro Intestinal diseases | 1 (4) | 0 (0) |
| **Single/multi-center** |  |  |
| Single center trial | 25 (96) | 29 (91) |
| Multi-center trial | 1 (4) | 3 (9) |
| **Use of a commercial BMD** |  |  |
| Yes | 21 (81) | 26 (81) |
| No | 5 (19) | 5 (16) |
| Unknown | 0 (0) | 1 (3) |
| **Funding** |  |  |
| Non-profit (Government, Universities, Non-profit NGO) | 19 (78) | 21 (66) |
| For-profit (Pharmaceutical industries) | 5 (13) | 3 (9) |
| Mixed | 1 (4) | 4 (13) |
| Not reported | 1 (4) | 4 (13) |

^1^ Many of these BMDs were used in addition to a smartphone application.

^2^ Percentages may not equal 100% due to rounding.
